# Plant-based dietary index in relation to gut microbiota in Arab women

**DOI:** 10.1097/MD.0000000000035262

**Published:** 2023-09-22

**Authors:** Ghadeer S. Aljuraiban, Esra’a A. Aljazairy, Abdulaziz S. Alsahli, Shaun Sabico, Sara Al-Musharaf

**Affiliations:** a Department of Community Health Sciences, College of Applied Medical Sciences, King Saud University, Riyadh, Saudi Arabia; b Department of Clinical Laboratory Sciences, College of Applied Medical Sciences, King Saud University, Riyadh, Saudi Arabia; c Chair for Biomarkers of Chronic Diseases, Biochemistry Department, College of Science, King Saud University, Riyadh, Saudi Arabia.

**Keywords:** dietary patterns, gastrointestinal microbiota, gut microbiome, plant-based diet, species

## Abstract

Plant-based foods may influence gut microbiota profiles and contribute to overall human health. However, not all plant-based diets are nutritionally equivalent. We aimed to assess the association between a plant-based dietary index (PDI), specifically unhealthy PDI and healthy PDI (hPDI), and gut microbial composition and diversity in young women in Saudi Arabia. This observational study included 92 healthy women aged 18 to 25 years. Dietary and anthropometric data were collected. Fecal samples were analyzed using a novel whole-genome shotgun sequencing technique. Alpha and beta diversities measured the richness and composition of the gastrointestinal system. Relationships were examined with Pearson correlation, linear regression, and Wilcoxon Rank-Sum tests. Participants with higher PDI had higher levels of *Bacteroides_u_s* than those with lower PDI. hPDI was positively correlated with *Bifidobacterium pseudocatenulatum, Bifidobacterium longum, Oscillibacter*, and *Lactobacillus acidophilus* and inversely correlated with *Clostridioides difficile (P* < .05). Unhealthy plant-based dietary index was inversely correlated with *B pseudocatenulatum, B longum*, and *L acidophilus* and positively correlated with *C difficile (P* < .05) and other species of interest. In conclusion, hPDI scores were significantly associated with microbiota species linked with favorable health outcomes, independent of body mass index and gut microbial richness and composition in Arab women. Future studies should investigate the modulating effect of plant-based diets on the species identified in the current study.

## 1. Introduction

Healthy plant-based dietary patterns are beneficial to human health, with epidemiological evidence supporting a link between plant-based diets (such as vegetables, fruits, whole grains, legumes, seeds, and nuts) and a reduced risk of several adverse health outcomes, such as cardiometabolic diseases.^[1–3]^ Although many potential mechanisms link plant-based diets with health outcomes, one interesting area of study is the contribution of gut microbiota, a collective term for microorganisms living in the gastrointestinal tract,^[4]^ which are essential for maintaining metabolic health.^[5,6]^ Major groups of microorganisms dominate the gut, nonetheless, variations in their species and abundance differ considerably.^[7]^ The healthy gut microbiota is dominated by the Bacteroidetes phylum, Firmicutes phylum, and smaller amounts of other phyla.^[8]^ Much of the diversity in the gut microbiota among people is associated with the Firmicutes/Bacteroidetes ratio (F: B ratio).^[9]^ Evidence suggests that the gut microbiota of individuals with cardiometabolic diseases (e.g., obesity or type 2 diabetes) differs from those without these conditions, potentially altering how energy and nutrients are utilized.^[10]^

Plant-based food intake may influence intestinal microbial profiles. A vegetarian, vegan, or healthy plant-based diet appears to result in differing gut compositions compared with diets with a higher intake of animal-based foods.^[11,12]^ In general, plant-based diets may promote a diverse gut ecosystem with greater levels of bacterial species that can benefit overall health.^[11,12]^ There is also some evidence that plant-based dietary interventions lead to a modest change in gut microbiota composition.^[13]^ As calls to transition to plant food sources for better community health and food sustainability are gaining traction,^[14]^ the potential of these diets to affect the gut microbiota has become increasingly important to examine; however, not all plant-based diets are nutritionally equivalent. Studies have demonstrated that the relationship between plant-based diets and health is mediated by the healthfulness of the diets rather than the source of the foods alone (i.e., plant vs animal). For instance, healthy plant-based diets (e.g., fruits, vegetables, legumes, nuts, whole grains) seem to have greater benefits for reducing the risk of several health outcomes and mortality than less healthful plant-based diets (e.g., refined grains, potatoes, juice).^[1–3,15–17]^ As such, the nuances of plant-based diets as they relate to the gut microbiota should be considered in investigations. Furthermore, research findings related to the gut microbiota from 1 group may not be generalizable to another group.^[18]^ Therefore, geography and population-specific cohorts are needed to examine gut microbiota patterns.^[19]^

This study aimed to assess the relationships between an overall plant-based dietary index (PDI), unhealthy plant-based dietary index (uPDI), healthy plant-based dietary index (hPDI), and gut microbial composition and diversity in young women in Saudi Arabia. We hypothesized that healthy plant-based diets, but not necessarily unhealthy plant-based diets, would be associated with gut microbiome characteristics that have been linked to positive health outcomes.

## 2. Materials and methods

### 2.1. Study design

This study was derived from a previous case-control study that identified gut composition in relation to obesity indices.^[20]^ Detailed methods on this cohort have been previously published^.[20]^ Briefly, between January 2019 and March 2020, Saudi female college students aged 18 to 25 years at King Saud University with obesity (body mass index [BMI] ≥ 30 kg/m^2^) and normal weight (BMI = 18.5–24.9 kg/m^2^) were recruited. Those who were overweight (BMI = 25.0–29.9 kg/m^2^), following a specific diet, taking vitamin B12, multivitamins, or antibiotics within the 6 months before the sample collection date, or with reported gastrointestinal diseases and other diseases (e.g., anorexia) were excluded. After screening, 92 Saudi females consented to participate in this study. Appointments were given to each participant to visit the study clinic and to provide demographic data, anthropometric parameters (height, weight, and hip/waist circumference), and body composition data. The local Institutional Review Board Committee of King Saud University approved this study (IRB #E-19-3625) in accordance with the principles of the Declaration of Helsinki.

#### 2.1.1. Anthropometric parameters.

Trained clinical dietitians collected anthropometric data using standardized methods. Measurements were recorded twice for each parameter. The mean of the 2 readings was used in the final analysis. With no shoes and light clothing, the participant’s weight and height were recorded to the nearest 0.1 kg and 0.5 cm, respectively. Weight measurements were taken using a Digital Pearson Scale (ADAM Equipment Inc., Oxford, CT). BMI was calculated by dividing weight (kilograms) by height (meters squared). Using a non-stretchable tape, waist circumference (the narrowest zone between the umbilicus and the lower rib) and hip circumference (at the trochanter level) were measured. Values were rounded to the nearest 0.5 centimeters. A third measurement was performed when there was a variation of > 2 cm between measurements. The 2 measures with the least variation were used. The mean waist circumference was divided against the mean hip circumference to obtain the waist-to-hip ratio (WHR).^[21]^ The cutoff value for WHR was 0.83 ( ≥ 0.83 was considered high) WHR.^[22]^ Bioelectrical impedance analysis (770 bioelectrical impedance analysis; InBody, Seoul, South Korea)^[23]^ was used to determine body composition, which provided data on; % body fat, % muscle mass, and % body water.

#### 2.1.2. Dietary data.

Dietary data were collected by a clinical dietitian using the Food and Drug Authority Food Frequency Questionnaire.^[24]^ The questionnaire consisted of 133 items and inquired about factors such as intake of salt, visible fat, and cooking methods.^[24]^ To estimate portion sizes as accurately as possible, photos, food modules, and household measurements were used during the interviews. The ESHA Food Processor Nutrition Analysis Software (ESHA Research Inc., version 10.8, 2010, Salem, Oregon) was used to analyze the dietary data. The software calculates each individuals total kcal intake (kcal/ day) and the macronutrient composition as (% of total calories) for carbohydrate, protein, and fat. The software also calculates average intake of food groups such as whole grains, fruits, vegetables, nuts, etc, presented in (g/1000 kcal). Recipes of mixed dishes and traditional Saudi foods were manually added to the software. Daily food intake was calculated by taking the total average intake frequency multiplied by the portion size.^[25]^

### 2.2. Plant-based diet indices

The PDI^[1]^ assigns positive points to plant-based foods in the diet and negative points to animal-based foods. It can then be used to further categorize intakes into hPDI and uPDI in which the PDI is differentiated according to nutritional contribution. For the hPDI, points are allocated for healthier plant-based foods (e.g., fruits, nuts, legumes, whole grains, tea, coffee, and vegetable oils) and taken away for less healthy plant-based foods (e.g., desserts, sweets, refined grains, sugar-sweetened beverages, and fruit juices). The opposite scoring system is used for uPDI. Animal foods (e.g., total meat, fish, eggs, and dairy products), animal fat (e.g., butter), and miscellaneous foods (e.g., hamburgers) are awarded negative points for PDI, hPDI, and uPDI. (see Table S1, Supplemental Digital Content, http://links.lww.com/MD/J914, which shows the definition of each score).

Participants were divided into tertiles of intake for each food item. With positive points, intakes equivalent to or above the Dietary Guidelines recommendations^[26]^ were assigned a score of 3 as they were considered the highest (maximum) tertile, wheras the score of 1 was considered the lowest. This scoring system was inversed with reverse scores. For example, when calculating PDI and uPDI, individuals in the highest tertile of sugary beverage consumption (a less healthy food) were given a score of 3, and those in the lowest ( < 1 c/d) were assigned a score of 1. The possible range of scores were 16 to 48 for the PDI, hPDI, and uPDI. A final score was calculated for each of the 3 indices (i.e., PDI, hPDI, uPDI). For any of the 3 measures, a high score was associated with a low consumption of animal-based foods.

### 2.3. Stool analysis

Dry containers were used to collect fecal samples, which were stored aseptically at − 80°C. Using a QIAamp PowerFecal DNA Isolation Kit (Qiagen, Hilden, Germany), DNA was extracted from 0.25 g samples of the stool. C6 elution buffer (100 μL) was used to elute the samples as described by the manufacturer. The concentration and purity (260/280 ratio) of the extracted DNA were measured using a NanoDrop spectrophotometer (NanoDrop Technologies). The DNA was stored at − 20°C until processing for library preparation and sequencing. The Illumina Nextera XT Library Preparation Kit (Illumina, Inc., San Diego, CA) was used to prepare DNA libraries using a modified protocol, and quantitative assessment was performed using a Qubit fluorimeter (Thermo Fisher Scientific, Milan, Italy). After library preparation, samples (2 × 150 bp) were sequenced using an Illumina sequencer.

### 2.4. Characterization of microbial composition

Whole-genome shotgun sequencing (WGS) analyses were to generate unrestricted genome sequencing for all the microorganisms present.^[27]^ This technique is used to sequence the human genome and is accomplished by unrestricted sequencing of the genomes of all the microorganisms present.^[27]^ It can help determine the gut microbial composition at the level of major microbial phyla. This technique improves the accuracy of preexisting sequence data, and corrects errors of other DNA sequencing methods.^[28]^ The Cosmos ID bioinformatics platform (Cosmos ID, Inc., Rockville, MD) was used to directly analyze the multi-kingdom microbiome for virulence genes and antibiotic resistance for all unassembled sequencing reads. This approach uses algorithms to mine data and databases with curated genomes to disambiguate numerous metagenomic sequences into discrete rendering sequences for microorganisms.

### 2.5. Statistical analysis

IBM SPSS Statistics for Windows (version 24; IBM Corp., Armonk, NY) was used for all the statistical analyses. The distribution of all quantitative variables was tested for normality before analyses. The median PDI score (median PDI = 43) was used to identify high and low PDI for descriptive analyses using a *t* test and results are presented as (mean [SD]).

Pearson correlation test was used to identify the correlations between plant-based indices (i.e., PDI, hPDI, uPDI) and gut microbiota composition. Associations between various taxonomic levels of gut microbes and plant-based indices were identified using a linear regression analysis.

To measure the gut microbiota richness and composition, we used alpha and beta diversity measures. Alpha diversity measures the diversity of the species in a specific community and is expressed as the number of species present, providing information on the species richness.^[29]^ Beta diversity is used to identify the change in species-diversity between communities and allows for the comparison between communities/ecosystems.^[29]^ Alpha and beta diversity measures were determined using the Cosmos ID taxonomic analysis (R software Vegan package, version 2.5–6, Oksanen et al^[30]^ 2019). Subsequently, the Wilcoxon Rank-Sum test was used to assess the statistical difference in alpha diversity between the high and low PDI indices, followed by plot generation using the same software.^[31]^ PERMANOVA was used to determine the difference in beta diversity between low and high PDI indices. The test was based on the Bray–Curtis distance using Vegan function adonis2. The ggpubr package, an R package used to provide visual plots, was applied. The Principal Coordinate Analysis Plot A function in the ggpubr package was used to generate plots.^[30,32]^ A *P* value < .5 was cosidered statistically significant.

The sample size was calculated based on previous literature^[33]^ that aimed to identify the composition of gut microbiota and associated differences based on a 5% significance level and 80% power. For an F:B of 0.9 ± 0.4 in females with normal weight and 1.7 ± 1.7 in those with obesity, the total sample size was n = 92. This sample size was comparable to previous published studies.^[34,35]^

## 3. Results

### 3.1. Descriptive characteristics

The study included 92 female college students. Descriptive data and gut composition by PDI score are summarized in (Table S2, Supplemental Digital Content, http://links.lww.com/MD/J916, which illustrates characteristics and gut composition stratified by PDI score).

Although no variation was observed in BMI, participants with a higher PDI score had lower WHR, energy intake, protein intake, and fat intake than those with a lower PDI score. Regarding food groups, those with higher PDI had more intake of whole grains and fruits and a lower intake of fruit juice, potatoes, sweets and desserts, eggs, and meat. For gut composition, participants with higher PDI had lower *Flavonifractor plautii, Clostridium bolteae, Bacteroides_u_s, Bacteria_u_p, Lactobacillus_acidophilus, Verrucomicrobia, Fusobacteria, and higher Clostridioides difficile, Bifidobacterium_kashiwanohense, Proteobacteria,* and F: B ratios than those with lower PDI (*P* < .001) (Table S2, Supplemental Digital Content, http://links.lww.com/MD/J916).

### 3.2. The relation between PDI scores and gut microbiota

hPDI was positively correlated with *Bifidobacterium pseudocatenulatum* and *Bifidobacterium longum*, whereas uPDI was inversely correlated with these same species Table 1. hPDI was inversely correlated with *C difficile*, positively correlated with *Lactobacillus acidophilus*, whereas uPDI was inversely correlated with *L acidophilus* (Table 1).

**Table 1:** Correlation between PDI scores, energy, body composition, and gut microbiota, n = 92.

	PDI	*P* value	hPDI	*P* value	uPDI	*P* value
Energy	−0.18	.08	0.11	.31	0.20	.06
BMI	−0.12	.25	−0.20	.06	0.24	.02
WHR	−0.06	.57	−0.03	.79	−0.01	.94
Waist	−0.12	.25	−0.15	.16	0.15	.16
Fat%	−0.15	.15	−0.12	.24	0.19	.07
Firmicutes	0.01	.98	0.08	.46	−0.04	.68
*Blautia wexlerae*	0.07	.52	0.01	.94	0.07	.53
*Flavonifractor plautii*	−0.03	.76	−0.01	.90	−0.08	.42
*Clostridium bolteae*	−0.08	.43	−0.13	.21	0.02	.82
*Faecalibacterium prausnitzii*	−0.12	.26	0.12	.26	0.01	1.00
*Clostridioides difficile*	0.16	.12	−0.20	.05	0.28	.01
Bacteroidetes	−0.02	.85	−0.06	.54	0.03	.80
*Bacteroides faecichinchillae*	−0.01	.92	−0.10	.32	0.15	.17
*Bacteroides thetaiotaomicron*	−0.04	.74	−0.09	.39	0.05	.67
*Bacteroides_u_s*	0.22	.03	−0.05	.02	−0.04	.67
*Bacteria_u_p*	−0.07	.52	0.03	.75	−0.02	.82
*Lactobacillus acidophilus*	0.15	.16	0.21	.05	−0.25	.02
Actinobacteria	0.08	.43	0.06	0.58	0.02	.84
*Bifidobacterium pseudocatenulatum*	0.05	.61	0.21	.03	−0.25	.02
*Bifidobacterium kashiwanohense*	−0.01	.95	−0.08	.43	0.01	.92
*Bifidobacterium longum*	0.27	.27	0.41	.02	−0.22	.05
Verrucomicrobia	0.02	.87	−0.10	.33	0.07	.52
Proteobacteria	0.01	.97	0.01	.94	−0.07	.50
Fusobacteria	−0.07	.50	−0.21	.05	0.09	.38
F: B ratio	0.04	.73	0.11	.29	−0.08	.42

BMI = body mass index, F: B = Firmicutes/Bacteroidetes, hPDI = healthy plant-based dietary index, PDI = plant-based dietary index, uPDI = unhealthy plant-based dietary index, WHR = waist-to-hip ratio.

**Figure 1.**
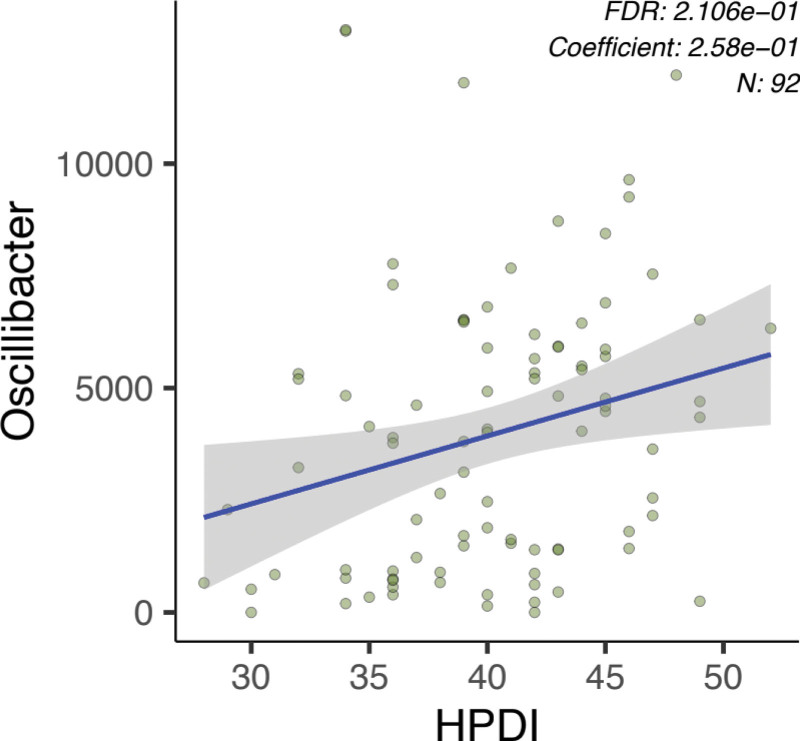
Linear regression analysis of Oscillibacter and a healthy plant-based dietary index (hPDI).

**Figure 2.**
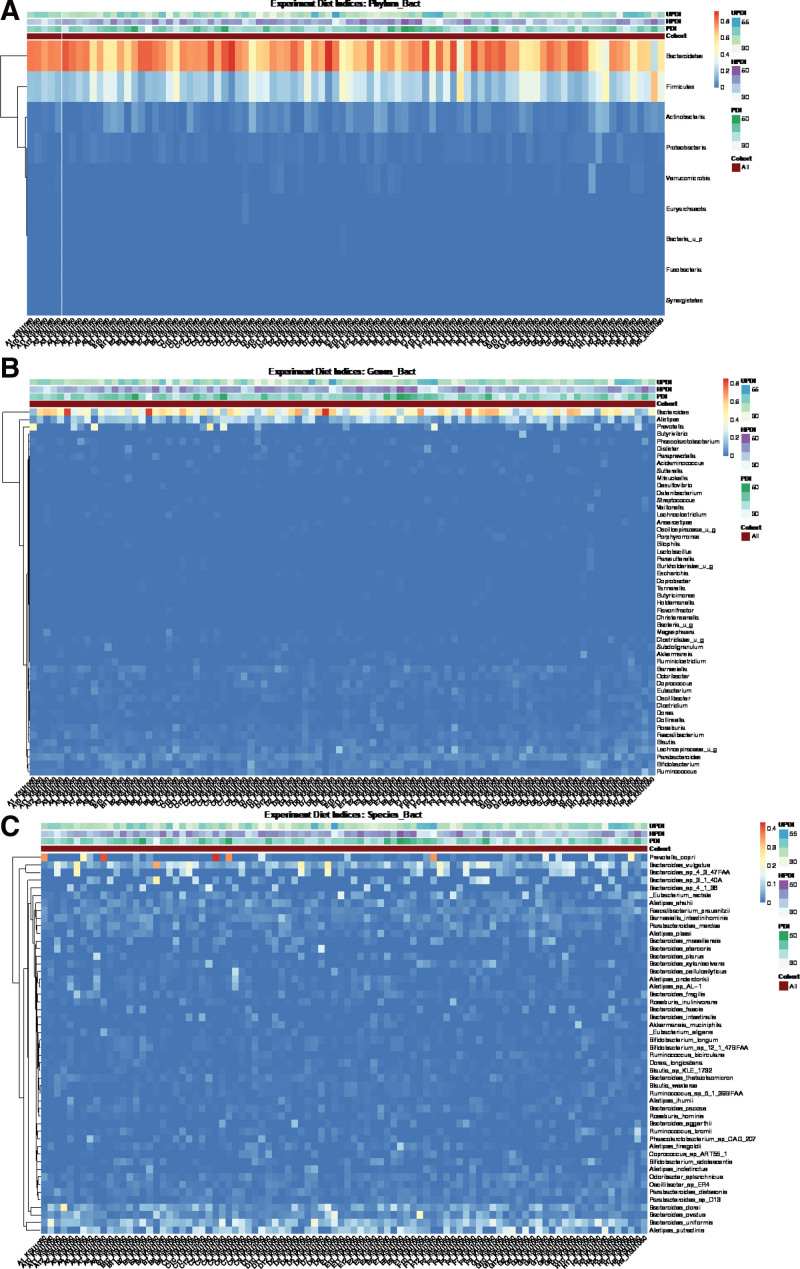
(A) Heatmap of the phyla associated with plant-based dietary indices, (B) Heatmap of the genera associated with plant-based dietary indices, (C) Heatmap of the species that associated with plant-based dietary indices.

Regression analysis revealed a significant positive correlation between the abundance of *Oscillibacter* and hPDI (Fig. 1). Hierarchical clustering of the heatmap showed no clear association between PDI and the gut microbiota at different taxonomic levels (Fig. 2). However, samples were dominated by high levels of *Bacteroides* phyla (Fig. 2A), *Bacteroides* genera (Fig. 2B) and *Prevotella copri, Bacteroides vulgatus, Bacteroides dorei, Bacteroides ovatus, Bacteroides uniformis, Alistipes putrescens*, and several unnamed *Bacteroides* species (Fig. 2C).

### 3.3. Alpha and beta diversity

**Figure 3.**
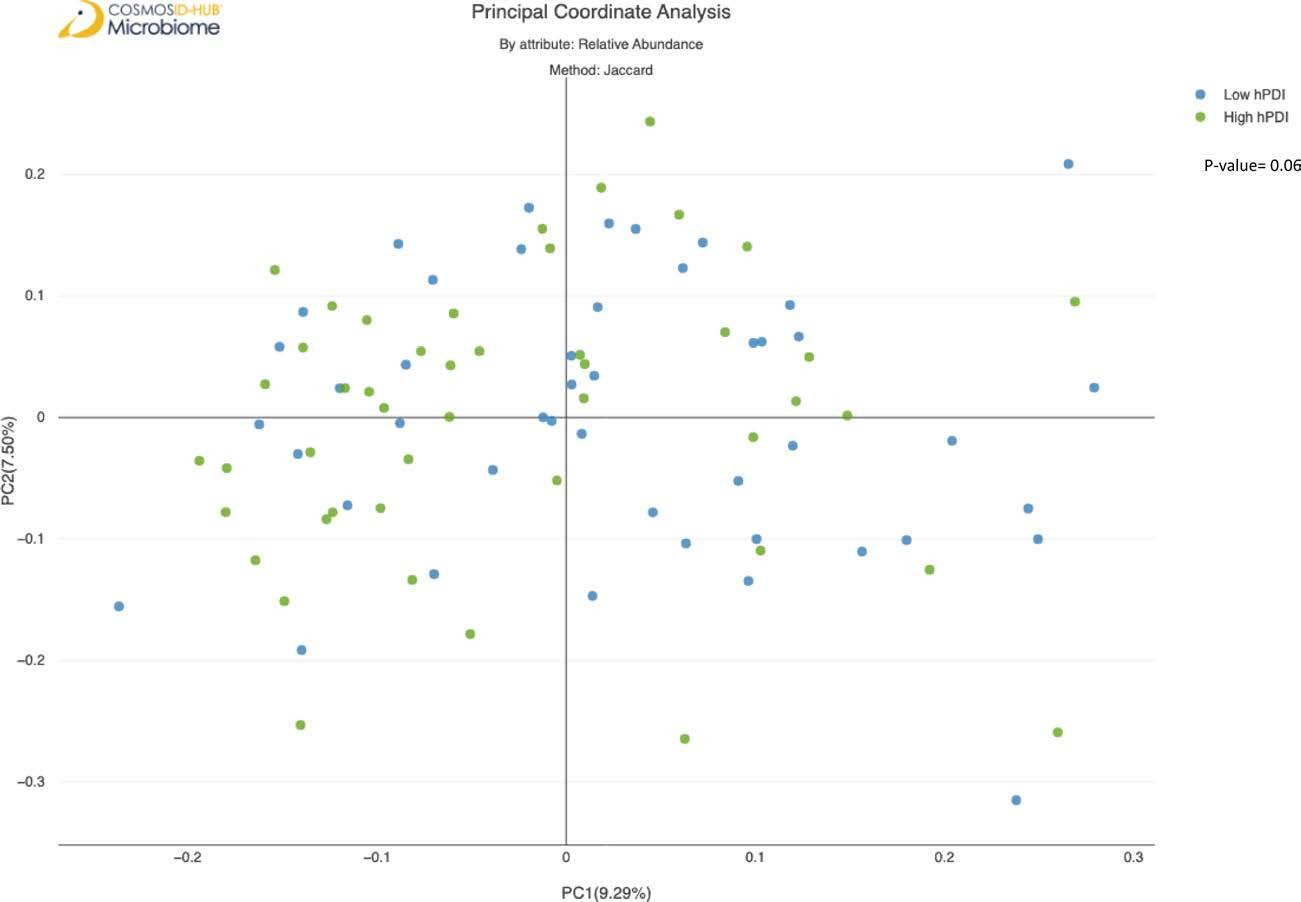
Beta diversity of gut microbiota of participants with high healthy plant-based dietary index (hPDI) compared to those with low hPDI.

Microbial alpha diversity, based on the Shannon index, was not significantly associated with PDI, hPDI, or uPDI (see Figure S1, Supplemental Digital Content, http://links.lww.com/MD/J917, which demonstrates Gut microbiota Alpha diversity of (a) PDI, (b) hPDI, (c) uPDI). According to Principal Coordinate Analysis Plot A, there was an association between microbial beta diversity and hPDI (*P* = .06) (Fig. 3). However, this association was not statistically significant.

## 4. Discussion

This study investigated the association between plant-based dietary indices and gut microbial composition and diversity in young women in Saudi Arabia. Results demonstrate that the association between microbial species and healthfulness of plant-based dietary indices vary. For some microbial species, the relationships were inversed for hPDI and uPDI, emphasizing the distinction between plant-based diets of varying nutritional compositions.

The association between gut microbiota and plant-based diets is biologically plausible. Healthy plant-based diets include vegetables, fruits, tea/coffees, nuts, legumes, and vegetable oils; and many of these foods contribute to healthy prebiotics such as fiber and lignans.^[36]^ Prebiotics are selectively fermented in the gastrointestinal tract and can influence the composition and activity of the gut microbiome to confer benefits to the host.^[36,37]^ For PDI scores in general, participants with a higher PDI had lower levels of several phyla, including a lower F: B ratio than those with a lower PDI. This is in line with research showing that vegetarian and vegan diets are associated with more *Bacteroidetes* and fewer *Firmicutes* than omnivorous diets.^[38]^

hPDI in the present study was positively correlated with several microbial strains associated with favorable health outcomes. For instance, the *Bifidobacterium* strain has shown favorable effects on body fat distribution.^[39]^ *B longum* and *B pseudocatenulatum* have been used in interventions to address obesity, reduce serum glucose, circulating insulin, and plasma triglycerides, and improve glucose tolerance in rat models.^[40–42]^ Additionally, supplementation with *Lactobacilli* (i.e., *Lactobacillus plantarum, L acidophilus*) and *Bifidobacteria* (i.e., *Bifidobacterium bifidum, Bifidobacterium animalis*) for 6 months led to a reduction in body weight, small dense LDL cholesterol, and an improved lifestyle in 220 Bulgarian adults with overweight or obesity.^[43]^ A recent study revealed a decline in neoplastic lesions in the colon with *Oscillibacter* in mice transplanted with rice-bran-modified microbiota.^[44]^ *Oscillibacter* is causally linked to decreased triglyceride concentrations in a Chinese population.^[45]^ Furthermore, *C difficile,* which was inversely correlated with hPDI and postively correlated with uPDI, is a pathogen known to cause diarrhea leading to substantial morbidity and mortality.^[46]^ These findings support the postulation that hPDI would be associated with beneficial microbial strains; while at the same time, many of these same strains were inversely related to uPDI, emphasizing the need to distinguish between the nutritional quality of plant-based diets.

While this is not the first study to examine the relationship between PDI and the gut microbiome, it is the first to do so in a cohort of young Saudi Arabian females. As such, the specific strains associated with PDI, hPDI, uPDI, are likely to differ from those ascertained in other populations. One similar study conducted in US males (n = 303), identified a positive association between hPDI and *Eubacterium eligens*, and *Bacteroides cellulosilyticus* abundance and a negative association with *Lachnospiraceae bacterium, Ruminococcus Torques, Ruminococcus gnavus, Clostridium leptum,* and *Erysipelotrichaceae bacterium*.^[12]^

In the present study, neither alpha nor beta microbial diversity was significantly associated with PDI, hPDI, or uPDI. As such, richness and composition did not differ with the varying levels of dietary indices. This is in contrast to other works that suggest diversity is related to plant-based intakes.^[11]^ For instance, there was a study of 3096 participants from China that found that short-term adherence to an hPDI was associated with microbial alpha diversity.^[47]^ There are several ways to explain these contradictory findings. Firstly, different techniques for identifying the composition of the gut microbiota may lead to different conclusons; the present study used the gold-standard WGS whereas previous studies have used the 16S rRNA sequencing.^[47]^ Additionally, it is possible that the differences in microbial diversity between participants is limited due to similarities within the sample population itself. There are cohort, cultural, and geography-specific patterns in gut microbiota compositions that may result in likenesses within a group.^[18,48]^ Further, age and potentially sex play a role in regulating the gut microbiota and this study focused only on women within a very limited age range, perhaps narrowing the range of microbiome diversity within the sample.^[49,50]^ These factors may also be considered to explain why there was no association between BMI and alpha diversity seen in this work, which is consistent with research in a Saudi population,^[48]^ but inconsistent with research conducted elsewhere.^[33]^

The main strength of this study is the use of a predefined dietary index. PDI allows for direct comparisons with other studies as it can consistently define a plant-based diet between cohorts. Another strength is the use of the WGS method, which is considered more accurate and sensitive than other methods available.^[28]^ This study presents an important contribution to the field because identifying hPDI and uPDI accounts for the potential diversity in plant-based diets and the differing association that this diversity can have with human health. Results can inform future research that examines the impact of healthy and unhealthy plant-based diets on the gut microbiota.

This study had several limitations. First, young women who were considered to be overweight were excluded. Although this allows for a more distinct comparison between those with obesity and normal weight participants, there may be differences in the dietary composition of overweight individuals, which could facilitate more in-depth understanding of the relationship between plant-based diets and gut microbes. Second, there was an inherent bias in the dietary data collection. Specifically, food frequency questionnaires are subject to recall bias, although this is still considered a reliable tool for estimating habitual intake.^[51]^ Third, the study focused on a specific population of Saudi female college students; therefore, the findings cannot be generalized to a broader population. Although, choosing this particular cohort addressed the lack of generalizability from other cohorts to this 1. Finally, we could not determine causality or rule out potential confounding factors as this was an observational study. Future studies can be designed to address these limitations.

## 5. Conclusion

In conclusion, hPDI scores were significantly associated with specific microbiota species associated with favorable health outcomes, independent of BMI and gut microbial richness and composition in young Arab women. Future studies can investigate the modulating effect of plant-based diets of differing nutritional quality on the gut microbiome.

## Author contributions

**Conceptualization:** Ghadeer S. Aljuraiban, Abdulaziz Alsahli, Shaun Sabico, Sara Al-Musharaf.

**Data curation:** Ghadeer S. Aljuraiban, Esra’a A Aljazairy, Shaun Sabico.

**Formal analysis:** Ghadeer S. Aljuraiban, Esra’a A Aljazairy, Abdulaziz Alsahli, Sara Al-Musharaf.

**Funding acquisition:** Ghadeer S. Aljuraiban, Sara Al-Musharaf.

**Methodology:** Esra’a A Aljazairy, Abdulaziz Alsahli, Shaun Sabico.

**Supervision:** Shaun Sabico, Sara Al-Musharaf.

**Writing – original draft:** Ghadeer S. Aljuraiban, Sara Al-Musharaf.

**Writing – review & editing:** Shaun Sabico.

## Supplementary Material






