# Supplementary material for: Plant-based dietary index in relation to gut microbiota in Arab women
Source: Medicine (Baltimore). 2023 Sep 22;102(38):e35262. doi: 10.1097/MD.0000000000035262 (PMC10519475; doi:10.1097/MD.0000000000035262)
Supplement: Supplementary file 1 [file medi-102-e35262-s001.docx]

**Supplementary Table 1. Definition of the plant-based indices: PDI, hPDI, and uPDI ^a^**

| **Plant Food Groups** | **Description with examples** | **PDI** | **hPDI** | **uPDI** |
| --- | --- | --- | --- | --- |
| **Healthy** |  |  |  |  |
| Whole grains | Whole grain breakfast cereal, other cooked breakfast cereal, cooked oatmeal, dark bread, brown rice, other grains, bran, wheat germ, popcorn, local whole grain dishes | Positive points | Positive points | Reverse points |
| Fruits | Fresh fruits (e.g. grapes, prunes, bananas, cantaloupe, watermelon, apples, pears, oranges, grapefruit, strawberries, blueberries, peaches, apricots, dates and plums) and dried fruits (e.g. raisins). | Positive points | Positive points | Reverse points |
| Vegetables | Tomatoes, tomato juice, tomato sauce, broccoli, cabbage, cauliflower, Brussel’s sprouts, carrots, mixed vegetables, yellow/winter squash, eggplant, zucchini, sweet potatoes, spinach cooked, spinach raw, kale, iceberg /head lettuce, romaine or leaf lettuce, celery, mushrooms, beets, alfalfa sprouts, garlic, avocado and corn | Positive points | Positive points | Reverse points |
| Nuts | Nuts, peanut butter | Positive points | Positive points | Reverse points |
| Legumes | String beans, tofu, soybeans, beans, lentils, peas and lima beans, lentil soup, falafel, hummus. | Positive points | Positive points | Reverse points |
| Vegetable oils | Oil-based salad dressing, vegetable oil used for cooking | Positive points | Positive points | Reverse points |
| Tea and coffee | Tea, coffee, Arabic coffee, and decaffeinated coffee | Positive points | Positive points | Reverse points |
| **Less healthy** |  |  |  |  |
| Fruit juices | Apple juice, orange juice, grapefruit juice, all other fruit juices | Positive points | Reverse points | Positive points |
| Refined grains | Refined grain breakfast cereal, white bread, bagels, rolls, muffins, biscuits, white rice, pancakes, waffles, crackers, and pasta | Positive points | Reverse points | Positive points |
| Potatoes | French fries, baked and mashed potatoes, potato and corn chips | Positive points | Reverse points | Positive points |
| Sugary beverages | Colas with caffeine and sugar, colas without caffeine but with sugar, other carbonated beverages with sugar, noncarbonated fruit drinks with sugar | Positive points | Reverse points | Positive points |
| Sweets and desserts | Chocolates, candy bars, candy without chocolate, cookies (home-baked and ready-made), brownies, doughnuts, cake (home-baked and ready-made), sweet roll (home-baked and ready-made), pie (home-baked and readymade), jams, jellies, preserves, syrup and honey, cream caramel, Arabic sweets | Positive points | Reverse points | Positive points |
| **Animal Food Groups** |  |  |  |  |
| Dairy | Skim low fat milk, whole milk, cream, sour cream, buttermilk, ice cream, yogurt, cottage and ricotta cheese, cream cheese, feta cheese, other cheese | Reverse points | Reverse points | Reverse points |
| Egg | All fried, scrambled, omelettes, and boiled eggs | Reverse points | Reverse points | Reverse points |
| Fish and seafood | Canned tuna, dark meat fish, other fish, shrimp, lobster, scallops | Reverse points | Reverse points | Reverse points |
| Total meat | Chicken and turkey with skin, chicken and turkey without skin, beef hot dogs, processed meats, liver, hamburger, beef-lamb mixed dish, beef-lamb main dish | Reverse points | Reverse points | Reverse points |
| Miscellaneous animal-based foods | Pizza, stuffed pastry, samosa, cream soup, added butter/lard, mayonnaise and other creamy salad dressing, soup, gravy, and salad dressing | Reverse points | Reverse points | Reverse points |
| Animal fat | Butter added to food, butter or lard used for cooking | Reverse points | Reverse points | Reverse points |
| ^a^ Plant Based Diet Index (PDI), healthy PDI (hPDI), unhealthy PDI (uPDI). | | | | |
