# Supplementary material for: Plant-based dietary index in relation to gut microbiota in Arab women
Source: Medicine (Baltimore). 2023 Sep 22;102(38):e35262. doi: 10.1097/MD.0000000000035262 (PMC10519475; doi:10.1097/MD.0000000000035262)
Supplement: Supplementary file 2 [file medi-102-e35262-s002.docx]

| **Supplementary Table 2. Descriptive characteristics and gut composition of participants stratified by PDI score, n=92 ^1^** | | | |
| --- | --- | --- | --- |
|  | **PDI <43** | **PDI ≥ 43** | ***p*-Value** |
| *n* | 50 | 42 |  |
| PDI (median) | 38 | 43 |  |
| Age (Years) | 21.1 (1.5) | 21.0 (1.5) | 0.88 |
| **Anthropometric measurements** |  |  |  |
| BMI (kg/m^2^) | 28.3 (7.6) | 28.8 (8.5) | 0.77 |
| WHR (ratio) | 0.8 (0.1) | 0.7 (0.1) | **0.05** |
| Body fat (%) | 42.5 (9.0) | 42.6 (9.8) | 0.55 |
| Muscle mass (%) | 27.3 (7.7) | 29.7 (9.3) | 0.20 |
| Body water (%) | 49.5 (8.4) | 42.1 (7.1) | **<0.0001** |
| **Dietary intake** |  |  |  |
| Energy (kcal/day) | 4389 (737) | 3332 (1116) | **<0.0001** |
| Carbohydrates (%of total kcal) | 49 (9) | 54 (8) | 0.42 |
| Protein (%of total kcal) | 17 (5) | 14 (3) | **<0.0001** |
| Fat (%of total kcal) | 46 (22) | 42 (15) | **0.009** |
| **Components of the Plant Based Scores (g/1000 kcal)** | | |  |
| Healthy |  |  |  |
| Whole grains | 27 (18) | 29 (30) | **0.002** |
| Fruit | 160 (146) | 218 (200) | **<0.0001** |
| Vegetables (excluding potatoes) | 190 (165) | 199 (158) | 0.11 |
| Nuts | 9 (10) | 12 (11) | 0.36 |
| Legumes | 17 (16) | 24 (21) | 0.06 |
| Tea and coffee | 104 (128) | 116 (100) | 0.11 |
| Less healthy |  |  |  |
| Fruit juices | 36 (84) | 12 (20) | **<0.0001** |
| Refined grains | 111 (59) | 100 (60) | 0.89 |
| Potatoes | 21 (12) | 18 (20) | **0.002** |
| Sugary beverages | 32 (39) | 24 (39) | 0.98 |
| Sweets and desserts | 31 (19) | 19 (13) | **0.02** |
| Animal Food Groups |  |  |  |
| Dairy | 125 (80) | 99 (70) | 0.37 |
| Egg | 18 (17) | 11 (10) | **0.0005** |
| Fish and seafood | 12 (10) | 12 (12) | 0.22 |
| Meat | 53 (35) | 34 (21) | **0.0009** |
| Miscellaneous animal-based foods | 120 (70) | 99 (78) | 0.77 |
| hPDI | 39 (5) | 40 (6) | 0.06 |
| uPDI | 41 (5) | 39 (7) | 0.06 |
| **Gut composition** |  |  |  |
| **Firmicutes** | 0.2355 (0.1045) | 0.2354 (0.1054) | 0.94 |
| *Blautia wexlerae* | 0.00689 (0.00508) | 0.00770 (0.00620) | 0.18 |
| *Flavonifractor plautii* | 0.00114 (0.00178) | 0.00101 (0.000886) | **<0.0001** |
| *Clostridium bolteae* | 0.000970 (0.00217) | 0.000603 (0.000954) | **<0.0001** |
| *Faecalibacterium prausnitzii* | 0.0218 (0.0127) | 0.0206 (0.0126) | 0.97 |
| *Clostridioides difficile*^§^ | 0.000072 (0.000380) | 0.000156 (0.000567) | **0.008** |
| **Bacteroidetes** | 0.7007 (0.1269) | 0.7083 (0.1262) | 0.97 |
| *Bacteroides faecichinchillae*^§^ | 0.000075 (0.000321) | 0.000063 (0.000275) | 0.31 |
| *Bacteroides thetaiotaomicron* | 0.00831 (0.00741) | 0.00873 (0.00812) | 0.54 |
| *Bacteroides_u_s* | 0.00509 (0.00951) | 0.00315 (0.00412) | **<0.0001** |
| *Bacteria_u_p* | 0.000970 (0.00167) | 0.000527 (0.000802) | **<0.0001** |
| *Lactobacillus_acidophilus* | 8.80000 (0.000062) | 0.000060 (0.000280) | **<0.0001** |
| **Actinobacteria** | 0.0410 (0.0340) | 0.0362 (0.0311) | 0.56 |
| *Bifidobacterium pseudocatenulatum* | 0.00285 (0.00512) | 0.00273 (0.00606) | 0.26 |
| *Bifidobacterium_kashiwanohense* | 0.000561 (0.00130) | 0.00135 (0.00234) | **0.0002** |
| **Verrucomicrobia** | 0.00568 (0.0145) | 0.00368 (0.00627) | **<0.0001** |
| **Proteobacteria** | 0.0147 (0.0142) | 0.0149 (0.00915) | **0.005** |
| **Fusobacteria** | 0.000170 (0.00120) | 0.000012 (0.000080) | **<0.0001** |
| **F:B ratio** | 0.38 (0.26) | 0.39 (0.42) | **0.001** |
| ^1^Presented as mean (SD) stratified by median of Plant-based dietary index  Body mass index (BMI); Healthy plant-based dietary index (hPDI); Firmicutes/Bacteroidetes (F:B); Plant-based dietary index (PDI); Unhealthy plant-based dietary index (uPDI); Waist-to-hip-ratio (WHR) | | | |
