# Supplementary material for: Plant-based dietary index in relation to gut microbiota in Arab women
Source: Medicine (Baltimore). 2023 Sep 22;102(38):e35262. doi: 10.1097/MD.0000000000035262 (PMC10519475; doi:10.1097/MD.0000000000035262)
Supplement: Supplementary file 3 [file medi-102-e35262-s003.docx]

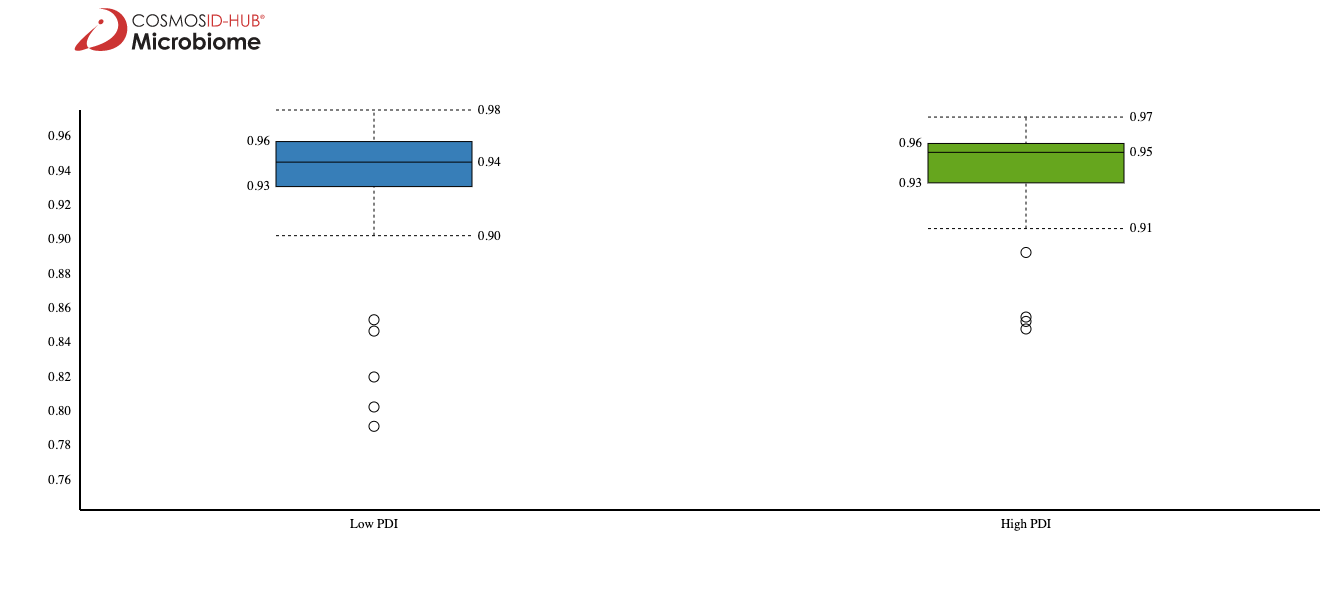


1. PDI


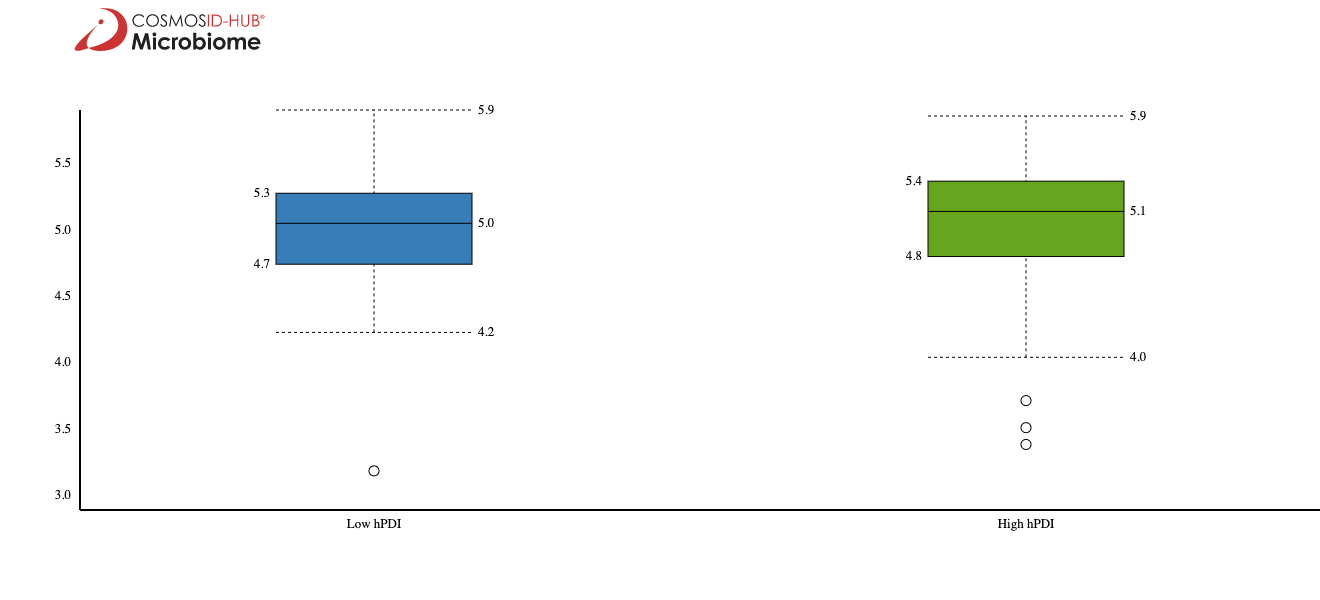


1. hPDI


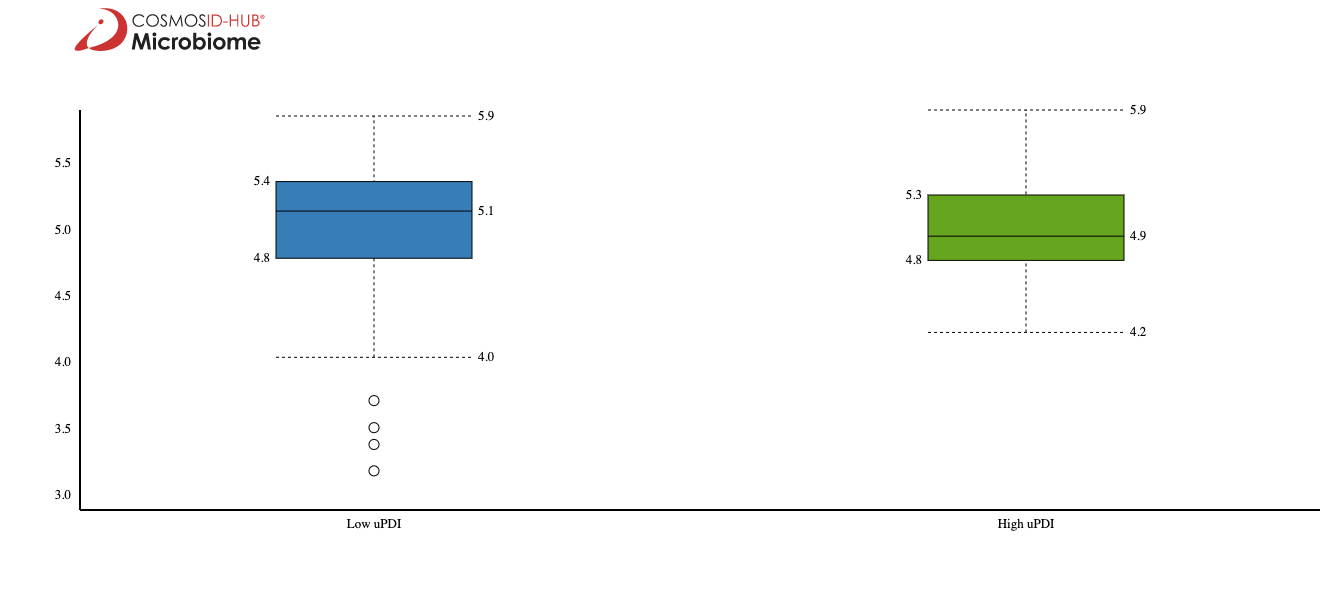


1. uPDI

**Supplementary figure 1. Gut microbiota Alpha diversity of plant-based dietary scores (PDI): (a) PDI, (b) healthy PDI (hPDI), (c) unhealthy PDI (uPDI)**
